# Novel Peptide Vaccine GV1001 Rescues Hearing in Kanamycin/Furosemide-Treated Mice

**DOI:** 10.3389/fncel.2018.00003

**Published:** 2018-01-19

**Authors:** Shin Hye Kim, Gaon Jung, Sangjae Kim, Ja-Won Koo

**Affiliations:** ^1^Department of Otorhinolaryngology-Head and Neck Surgery, Korea University Medical Center, Korea University College of Medicine, Seoul, South Korea; ^2^Department of Otorhinolaryngology-Head and Neck Surgery, Seoul National University Bundang Hospital, Seoul National University College of Medicine, Seongnam, South Korea; ^3^GemVax & Kael Co., Ltd, Seongnam, South Korea; ^4^Sensory Organ Research Institute, Seoul National University Medical Research Center, Seoul, South Korea

**Keywords:** aminoglycoside, kanamycin, furosemide, ototoxicity, GV1001, peptide vaccine, deaf

## Abstract

The cell-penetrating peptide GV1001 has been investigated as an anticancer agent and recently demonstrated anti-oxidant and anti-inflammatory effects. It has shown a protective effect on a kanamycin (KM)-induced ototoxicity mouse model. In the present study, we administered GV1001 at different time points after inducing hair cell damage, and examined if it rescues hair cell loss and restores hearing. A deaf mouse model was created by intraperitoneal injection of KM and furosemide. First, to test the early temporal change of hearing and extent of hair cell damage after KM and furosemide injection, hearing and outer hair cells (OHCs) morphology were evaluated on day 1, day 2 and day 3 after injection. In the second experiment, following KM and furosemide injection, GV1001, dexamethasone, or saline were given for three consecutive days at different time points: D0 group (days 0, 1, and 2), D1 group (days 1, 2, and 3), D3 group (days 3, 4, and 5) and D7 group (days 7, 8, and 9). The hearing thresholds were measured at 8, 16, and 32 kHz before ototoxic insult, and 7 days and 14 days after KM and furosemide injection. After 14 days, each turn of the cochlea was imaged to evaluate OHCs damage. GV1001-treated mice showed significantly less hearing loss and OHCs damage than the saline control group in the D0, D1 and D3 groups (*p* < 0.0167). However, there was no hearing restoration or intact hair cell in the D7 group. GV1001 protected against cochlear hair cell damage, and furthermore, delayed administration of GV1001 up to 3 days rescued hair cell damage and hearing loss in KM/furosemide-induced deaf mouse model.

## Introduction

Aminoglycoside antibiotics are used in the treatment of gram-negative bacterial infections, multi-drug resistant tuberculosis, and other hospital acquired serious infections. Dose-limiting side effects include cochlear and/or vestibular toxicity and nephrotoxicity. Aminoglycosides result in hair cell death by either caspase-dependent or -independent mechanisms (Rybak and Ramkumar, 2007). Aminoglycoside induced hair cells impairment by first inducing disarray of stereocilia and inflammatory changes in inner ear structures (Nakagawa et al., 1998; Cunningham et al., 2002; Kitahara et al., 2005; Tabuchi et al., 2007), ultimately terminating in apoptotic cell death through the formation of reactive oxygen species (ROS) including free radicals (Forge and Fradis, 1985; Priuska and Schacht, 1995).

A novel peptide vaccine GV1001, which is a cell-penetrating peptide (16-amino-acid sequence) derived from the active site of human telomerase reverse transcriptase (hTERT), has been investigated as an anticancer agent. GV1001 has been used against advanced pancreatic cancer, melanoma, non-small cell lung cancer, advanced hepatocellular carcinoma, cutaneous T-cell lymphoma and B-cell chronic lymphocytic leukemia. As an anticancer agent, GV1001 binds multiple human leukocyte antigen (HLA) class II molecules and elicits combined CD4 and CD8 T-cell responses (Kyte et al., 2011).

Inflammatory reactions, oxidative stress and apoptotic cell death were reportedly prevented by GV1001 delivered to the kidney and various cancer and primary blood cell lines (Bernhardt et al., 2006; Brunsvig et al., 2006; Lee et al., 2013; Koo et al., 2014). Recently, GV1001 also demonstrated cellular proliferation, stem cell mobilization, anti-apoptotic, anti-aging and anti-oxidant effects (Martínez and Blasco, 2011; Park et al., 2014). GV1001 exerts anti-inflammatory effects by inhibiting leukocyte migration and the release of pro-inflammatory cytokines, such as interleukin-6 (IL-6) and monocyte chemoattractant protein-1 (MCP-1; Koo et al., 2014). GV1001 may be an effective anti-inflammatory peptide that downregulates the production of pro-inflammatory cytokines through the suppression of p38 mitogen-activated protein kinase (MAPK) and nuclear factor (NF)-κB following Enolase1 (ENO1) stimulation (Choi et al., 2015). GV1001 blocks β-amyloid toxicity by mimicking the extra-telomeric functions of hTERT, consequently showing anti-apoptotic and anti-oxidant effects in rat neural stem cells (Park et al., 2014). GV1001 exerts a protective effect in skin flap of rat against ischemia-reperfusion injury through anti-oxidant effects, reducing ROS and suppressing the inflammatory cascade (Lee et al., 2017).

Most therapeutic trials given at the same time or prior to administration of aminoglycoside have claimed effective in protecting aminoglycoside ototoxicity (Song and Schacht, 1996; Song et al., 1998; Sha and Schacht, 1999; Himeno et al., 2002; Wang et al., 2012; Campbell et al., 2016). GV1001 also has shown a protective effect on a kanamycin (KM)-induced ototoxicity mouse model in our previous study (manuscript under review). The anti-inflammatory, anti-oxidant and anti-apoptotic effects of GV1001 seem to be effective on KM-induced cochlear damage. However, experimental preclinical trials reporting rescue effect after cochlear damage are very rare.

Ototoxic hair cell injury in a mouse can be made either by multiple injections of KM or a single injection of KM with furosemide (Hirose et al., 2014). Though multiple injection of KM would be ideal to make an animal model of aminoglycoside ototoxicity, it usually takes around 2 weeks to induce ototoxicity (Jansen et al., 2013). Other shortcomings are chance of losing mice over the course of multiple injections and, variable and unclear onset of hearing loss if hearing is not tested frequently (Hirose and Sato, 2011). On the contrary, a deaf mouse model induced by single injection of KM and furosemide would not only be easier to conduct animal experiments, but the onset of hearing loss and hair cell damage is also immediate after injection of KM and furosemide as seen in this experiment.

In the present study, we administered GV1001 at different time points after inducing hair cell damage, and examined if it rescues hair cell loss and restores hearing in a KM/furosemide-induced ototoxicity mouse model.

## Materials and Methods

### Deaf Mouse Model and Study Groups

A deaf mouse model (C57BL/6 mouse, 4–6 weeks of age, weight of 15–25 g) was created by intraperitoneal injection of KM (1000 mg/kg) followed by furosemide (100 mg/kg) within 30 min. The study protocols were approved by the Institutional Animal Care and Use Committee of Seoul National University Bundang Hospital (BA-1504-174-017). In Experiment 1, to assess the initial temporal change of hearing and the extent of hair cell damage in this deaf mouse model, total nine mice were divided into three groups: Day-1 (*N* = 3), Day-2 (*N* = 3) and Day-3 (*N* = 3). After injection of KM and furosemide on day 0, hearing loss and cochlear hair cell damage were evaluated on day 1, day 2 and day 3, respectively (Supplementary file S1).

In Experiment 2, to test the rescue effect of GV1001, total 120 mice were divided into the following three treatment groups: GV1001 (*N* = 40), dexamethasone (*N* = 40) and saline (*N* = 40). GV1001 (10 mg/kg; GemVax & Kael Co., Ltd, Seongnam, South Korea), dexamethasone (15 mg/kg), or saline was subcutaneously administered for three consecutive days after the injection of KM and furosemide. To compare the rescue effect of GV1001 on different time points, each group was divided into four subgroups according to the time points of GV1001, dexamethasone, and saline treatment: D0 group (days 0, 1 and 2), D1 group (days 1, 2 and 3), D3 group (days 3, 4 and 5), and D7 group (days 7, 8 and 9; Supplementary file S2).

### Assessment of Hearing Loss

All of the mice underwent an auditory brainstem response (ABR) test (SmartEP; Intelligent Hearing Systems, Miami, FL, USA) under intraperitoneal injection of ketamine (100 mg/kg) and xylazine (10 mg/kg) after inhalation of isoflurane. During the ABR test, a heating pad was applied to maintain body temperature. Tone burst (envelope, Blackman; duration, 1562 μs; stimulation rate, 21.1/s) stimuli at 8, 16 and 32 kHz were delivered to the external auditory meatus through plastic earphones connected to an EC1 electrostatic speaker. Subdermal needle electrodes were applied behind the ipsilateral mastoid (reference electrode), behind the contralateral mastoid (active electrode), and on the vertex (ground electrode).

The evoked responses were amplified, and 1024 sweeps were averaged in real time. To acquire auditory thresholds, the sound intensity of the tone burst stimuli was lowered by 10 dB intervals from 90 dB SPL. The auditory threshold was defined as the lowest sound intensity at which the most robust and stable component was evoked around 4 ms (Wave III; Scimemi et al., 2014).

### Tissue Preparation

Under anesthesia, venous blood was obtained before cardiac perfusion with PBS, followed by 4% paraformaldehyde (pH 7.4), and the cochlea and kidney were immersion-fixed (Koo et al., 2011). To prepare the cochlear whole-mount, the membranous labyrinth of the cochlea was dissected under a microscope and then fixed with 4% paraformaldehyde. Specimens were soaked in 0.3% Triton-X blocking solution for 1 h. Fixed tissues were labeled with Alexa 488-conjugated phalloidin for 1 h, washed, and then fixed with 4% paraformaldehyde. Specimens were mounted on slides with the anti-fade fluorescence mounting media VECTASHIELD^®^ (Vector Laboratories, Burlington, ON, Canada), and examined using confocal microscopy (Carl Zeiss MicroImaging, Oberkochen, Germany) under uniform 63× magnification for all imaging analyses.

Each row of outer hair cells (OHCs) was evaluated for the presence or absence of hair cells. OHCs were considered missing if there was a gap in the normal hair cell array or if there were no apparent stereocilia or cuticular plates. The number of morphologically intact OHCs was counted manually and averaged in five different fields. The percentage of morphologically intact OHCs, defined as “intact OHCs rate”, was calculated at each cochlear turn (apex/middle/base).

### Assessment of Nephrotoxicity

The mice were anesthetized, and venous blood was collected to measure blood urea nitrogen (BUN) values prior to trans-cardiac perfusion with saline and 4% paraformaldehyde fixative (pH 7.4). The BUN values were measured using Stat Profile^®^ Critical Care Xpress (Nova Biomedical, Waltham, MA, USA). The operating range for BUN was 6–102 mg/dL; the normal range of BUN in mice is 13–35 mg/dL (Stender et al., 2007).

Renal tubules were harvested and imaged by optical microscopy after hematoxylin and eosin (H&E) staining. The morphology was quantified as 0 (normal), 1 (areas of focal granulovacuolar epithelial cell degeneration and granular debris in the tubular lumens), 2 (tubular epithelial necrosis and desquamation seen easily but involving less than half of the cortical tubules), 3 (more than half of the proximal tubules showing desquamation and necrosis, and the involved tubules being found easily), and 4 (complete or almost complete tubular necrosis; Kapić et al., 2014). Ten consecutive fields were examined.

### Statistical Analysis

Statistical analyses were performed using SPSS for Windows (ver. 21; SPSS Inc., Chicago, IL, USA) and STATA v. 14.0 for Windows software (Stata Corp., College Station, TX, USA). The Kruskal-Wallis H test with Bonferroni correction were performed to compare continuous variables among the three groups and four subgroups: *p*-values less than 0.0167 or 0.0083 when compare three or four groups, respectively, were considered statistically significant.

## Results

### Early Temporal Change of Hearing and Cochlear Hair Cell after Kanamycin and Furosemide Injection

**Figure 1:**
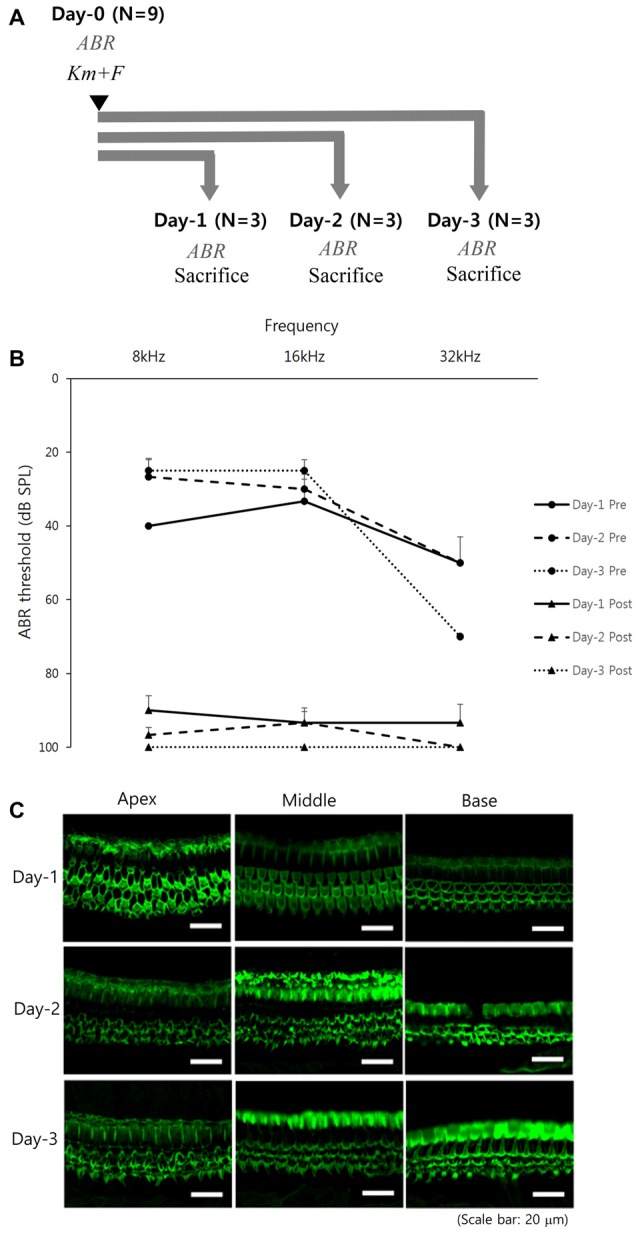
Early temporal change of hearing and cochlear hair cell after kanamycin (KM) and furosemide injection (Experiment 1): **(A)** study design, **(B)** hearing thresholds over time, and **(C)** hair cell damage among the Day-1 (*N* = 3), Day-2 (*N* = 3), and Day-3 (*N* = 2) groups. Error bars in auditory brainstem response (ABR) indicate the standard error of the mean (SEM).

From the Experiment 1 designed to assess the initial temporal change of hearing and the extent of hair cell damage (Figure 1A), the hearing was markedly deteriorated even from the next day (Day-1 group) of KM and furosemide injection (Figure 1B). However, OHCs morphology of apical, middle and basal turns were intact in two out of three mice of the Day-1 group. Three rows of the OHCs were completely disorganized and missed in the remaining 1 mouse of the Day-1 group and all five mice of the Day-2 and Day-3 groups (1 mouse of the Day-3 group died, Figure 1C; Supplementary file S3).

### Effect of GV1001 on Hearing Loss

**Figure 2:**
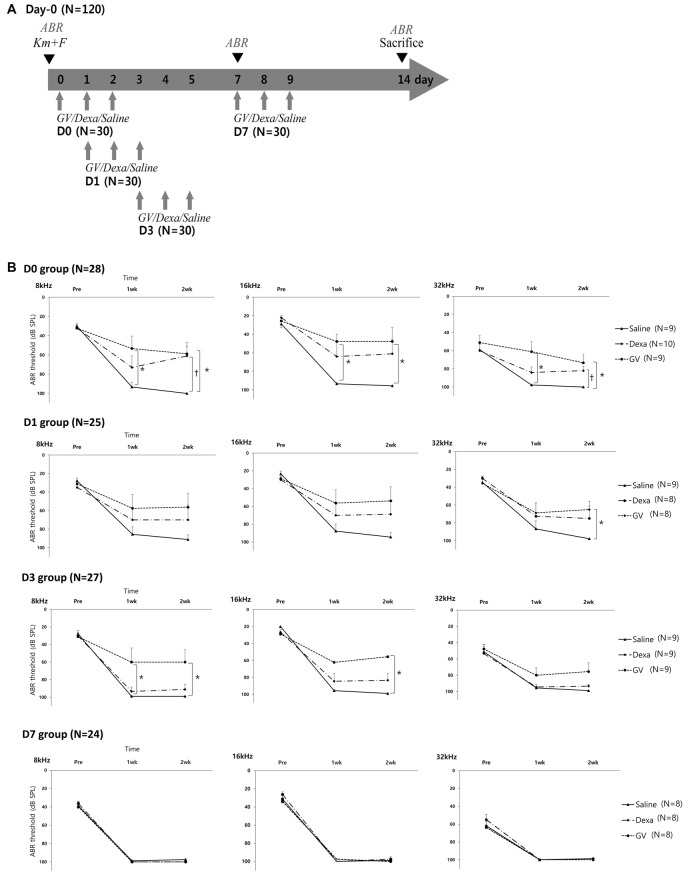
Effect of GV1001, dexamethasone and saline treatment in KM/furosemide-induced ototoxicity mouse model (Experiment 2): **(A)** study design, **(B)** hearing thresholds over time among the saline, dexamethasone and GV1001 groups. According to the time points of GV1001, dexamethasone, and saline treatment, all mice were classified as D0 (*N* = 28), D1 (*N* = 25), D3 (*N* = 27) and D7 (*N* = 24) subgroups. Error bars in auditory brainstem response (ABR) indicate the SEM. Statistically significant differences (*p* < 0.0167) are marked between saline-GV1001 groups (*) and saline-dexamethasone groups (†).

In the Experiment 2 designed to test the rescue effect of GV1001 (Figure 2A), the hearing thresholds at 8, 16 and 32 kHz over 2 weeks were compared among the three groups (GV1001, dexamethasone and saline) and four subgroups (D0, D1, D3 and D7; Figure 2B). After injection of KM and furosemide, the hearing thresholds increased in all three groups and all four subgroups. However, the hearing thresholds of the GV1001 group were relatively well preserved compared with those of the dexamethasone and saline groups with statistical significance (*p* < 0.0167).

Difference in the hearing thresholds between the GV1001 and saline control group was statistically significant at all six points in the D0 group: 8 kHz (*p* = 0.007), 16 kHz (*p* = 0.006), and 32 kHz (*p* = 0.001) at 1 week, and 8 kHz (*p* = 0.013), 16 kHz (*p* = 0.008), and 32 kHz (*p* = 0.003) at 2 weeks. In the D1 group, the significant difference between the GV1001 and saline control group showed at one points: 32 kHz (*p* = 0.014) at 2 weeks. In the D3 group, the significant difference between the GV1001 and saline control group showed at three points: 8 kHz (*p* = 0.006) at 1 week, and 8 kHz (*p* = 0.005) and 16 kHz (*p* = 0.001) at 2 weeks. On the other hand, a statistically significant difference to the saline control group, in the hearing thresholds of the dexamethasone group, was seen at two points in the only D0 group: 8 kHz (*p* = 0.007) and 32 kHz (*p* = 0.012) at 2 weeks. There was no statistically significant difference in the hearing thresholds between the GV1001 and dexamethasone group. In the D7 group, the hearing thresholds of all three groups were increased to nearly 100 dB SPL (complete hearing loss).

**Figure 3:**
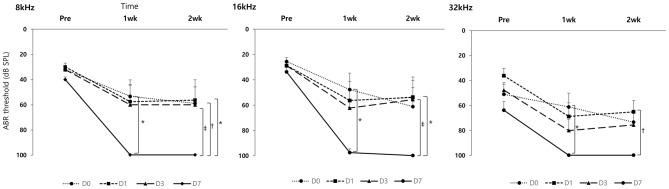
Rescue effect of GV1001 on hearing loss according to the time points of GV1001 treatment: hearing thresholds over time among the D0 (*N* = 9), D1 (*N* = 8), D3 (*N* = 9) and D7 (*N* = 8) subgroups in the GV1001 group. Error bars indicate the SEM. Statistically significant difference (*p* < 0.0083) is marked between D0–D7 groups (*), D1–D7 groups (†), and D3–D7 groups (‡).

In the GV1001 group, when compared the hearing thresholds among the D0, D1, D3 and D7 groups, the hearing thresholds of the D0, D1 and D3 groups were found to be statistical significantly different vs. that of the D7 group (*p* < 0.0083; Figure 3). In comparison between D0 and D7 group, hearing thresholds at four points, 8 kHz (*p* = 0.004), 16 kHz (*p* = 0.001), and 32 kHz (*p* = 0.001) at 1 week, and 16 kHz (*p* = 0.002) at 2 weeks, were significantly different. In comparison between D1 and D7 group, hearing thresholds at three points, 32 kHz (*p* = 0.003) at baseline, and 8 kHz (*p* = 0.004) and 32 kHz (*p* = 0.003) at 2 weeks, showed statistically significant difference. Moreover, in comparison between D3 and D7 group, hearing thresholds at one points, 8 kHz (*p* = 0.007) at 2 weeks, showed statistically significant difference. However, we found no significant difference in the hearing thresholds at any treatment time points among the D0, D1 and D3 groups (Supplementary file S4).

### Effect of GV1001 on Hair Cell Damage

**Figure 4:**
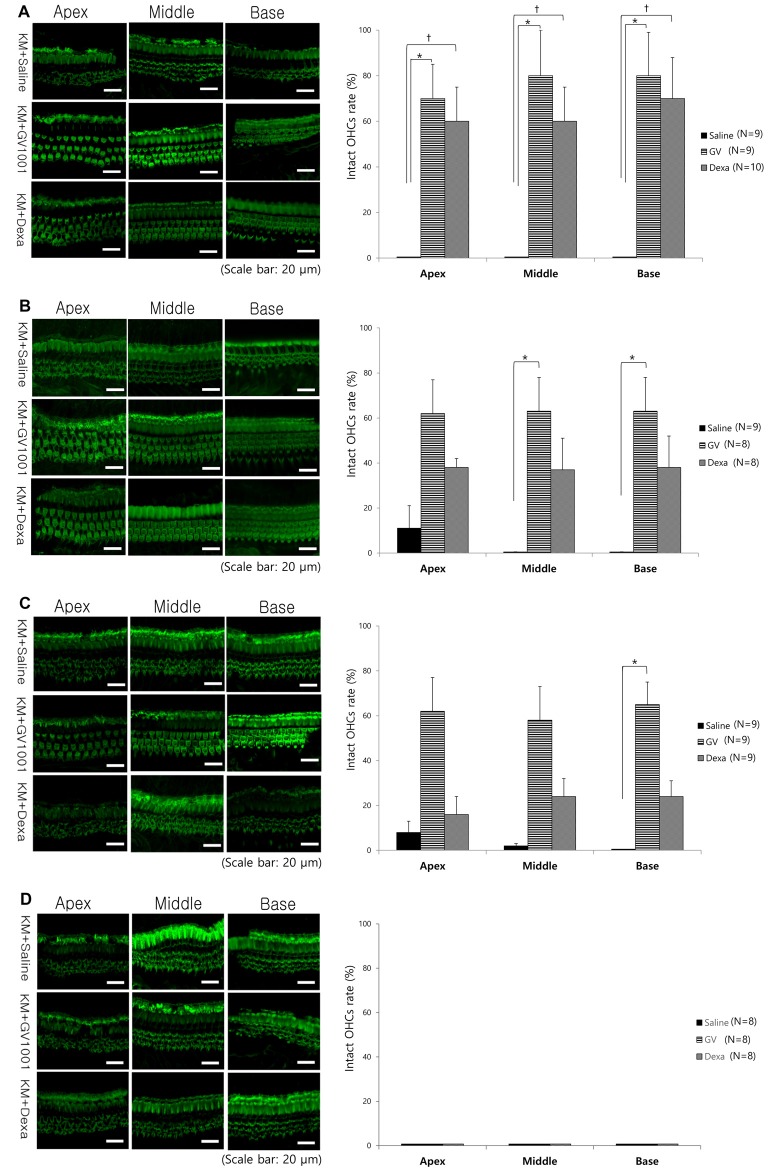
Effect of GV1001, dexamethasone and saline treatment on cochlear hair cell damage: hair cell damage among the saline, dexamethasone and GV1001 groups in **(A)** D0 (*N* = 28), **(B)** D1 (*N* = 25), **(C)** D3 (*N* = 27) and **(D)** D7 (*N* = 24) subgroups. Error bars indicate the SEM. Statistical significant difference (*p* < 0.0167) is marked between saline-GV1001 groups (*) and saline-dexamethasone groups (†).

Regarding the cochlear whole-mount results, almost all the OHCs in the saline group were lost or disorganized in all D0, D1, D3 and D7 groups (Figure 4). However, the count of morphologically intact hair cells were significantly higher in GV1001-treated mice than saline-treated mice in all D0, D1 and D3 groups, especially at the basal turns (*p* < 0.0167). In the D0 group (Figure 4A), the significant difference between the GV1001 and saline control group showed at all of the cochlear turns: apex (*p* = 0.005), middle (*p* = 0.002), and base (*p* = 0.003). In the D1 group (Figure 4B), the significant difference between the GV1001 and saline control group showed at the middle turn (*p* = 0.005) and basal turn (*p* = 0.007). In the D3 group (Figure 4C), the significant difference between the GV1001 and saline control group showed at the basal turn (*p* = 0.008). On the other hand, dexamethasone-treated mice showed a statistically significant intact OHCs morphology than the saline group at each cochlear turn only in the D0 group: apex (*p* = 0.010), middle (*p* = 0.009), and base (*p* = 0.002). However, the OHCs and hearing could not be rescued by dexamethasone or GV1001 when treated 7 days after injection of KM and furosemide (Figure 4D).

**Figure 5:**
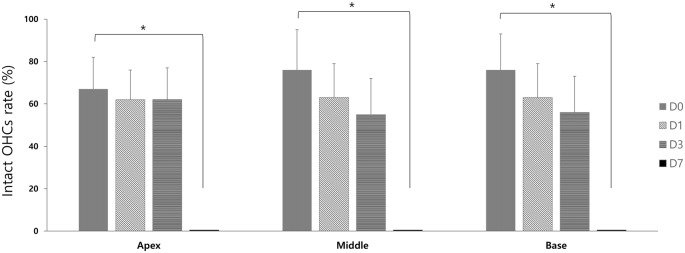
Rescue effect of GV1001 on hair cell damage according to the time points of GV1001 treatment: hair cell damage among the D0 (*N* = 9), D1 (*N* = 8), D3 (*N* = 9) and D7 (*N* = 8) subgroups in the GV1001 group. Error bars indicate the SEM. Statistical significant difference (*p* < 0.0083) is marked between D0–D7 groups (*). OHCs, outer hair cells.

In the GV1001 group, when compared hair cell damage among the D0, D1, D3 and D7 groups, the number of intact hair cells of the D0 group was significantly higher than that of the D7 group (*p* < 0.0083): apex (*p* = 0.005), middle (*p* = 0.003), and base (*p* = 0.005; Figure 5), while the comparison between the D0 group with D1 group or D3 group did not show statistical significance (Supplementary file S4).

### Effect of GV1001 on the Kidney

The BUN values of tested mice in the GV1001, dexamethasone, and saline groups ranged from 12 mg/dL to 33 (19.06 ± 4.77) mg/dL, and these values were within the normal range for BUN values in mice (8–33 mg/dL). Moreover, the renal tubules of all GV1001, dexamethasone, and saline treated mice showed normal morphology in the H&E staining. Thus, the administration of 10 mg/kg GV1001 was safe in mice and did not cause nephrotoxicity (Supplementary file S4).

## Discussion

In the present study, cell penetrating peptide GV1001 was tested if it rescues hair cell loss and restores hearing after inducing hair cell damage using KM/furosemide-induced deaf mouse model. We also tested if there is a therapeutic window to reverse hair cell damage by investigating agents at different time points of D0, D1, D3 and D7. The KM/furosemide-induced ototoxicity mouse model was ideal for this experiment since substantial hearing loss was documented on the next day of KM and furosemide injection, and complete loss of OHCs was seen on the second day of injection. GV1001-treated mice showed significantly less hearing loss and less hair cell damage than the saline control group in the D0, D1 and D3 subgroups. Therefore, delayed administration of GV1001 up to 3 days could rescue hearing loss and hair cell damage, while hearing loss was irreversible if it was given 7 days after ototoxic insult in KM/furosemide-induced deaf mouse model.

The potential otoprotective effects of several antioxidants have been tested in animal models. Iron chelators such as desferoxamine and dihydroxybenzoate (in guinea pig, Song and Schacht, 1996; Song et al., 1998) as well as antioxidants such as lipoic acid (in mouse, Wang et al., 2012), α-tocopherol (in guinea pig, Fetoni et al., 2004), D-methionine (in guinea pig, Campbell et al., 2016), and salicylates (in guinea pig, Sha and Schacht, 1999) successfully reduced the aminoglycoside-induced ototoxicity. Moreover, intra-cochlear administration of dexamethasone attenuated aminoglycoside-induced ototoxicity in the guinea pig (Himeno et al., 2002). In this study, GV1001-administered mice showed less hearing loss, with reduced hair cell damage, vs. the dexamethasone- and saline-treated mice even after the onset of hearing loss. The anti-inflammatory, anti-oxidant and anti-apoptotic effects of GV1001 may explain why GV1001 rescues cochlear hair cells against KM/furosemide-induced ototoxicity.

Clinically, there are numerous therapeutic approaches to treat ototoxicity, including antioxidants, ROS scavengers, apoptosis inhibitors, neuroprotective compounds and anti-inflammatory drugs such as steroids (Atar and Avraham, 2005; Rybak and Whitworth, 2005; Maruyama et al., 2008; Tabuchi et al., 2010; Mukherjea et al., 2011; Mizutari et al., 2013). The treatment of N-acetylcysteine (NAC) significantly reduced the ototoxicity in hemodialysis patients with gentamicin-induced hearing loss (Feldman et al., 2007). Use of aspirin significantly reduced the incidence of hearing loss in patients receiving gentamicin and aspirin when compared to the incidence in placebo group (3% vs. 13%; Sha et al., 2006). Our previous study proved that GV1001 (0.1–100 mg/kg) itself did not have any detrimental effects on the inner ear or kidney. As shown in Figures 2B, 4, the saline-treated mice showed complete hearing loss and damaged cellular framework of OHCs. The normal range of BUN values of all 40 mice in the saline group indicated that the increased ototoxicity in the saline group was not potentiated by nephrotoxicity.

As well known, OHCs damage caused by aminoglycoside-induced ototoxicity is most prominent at the basal turn of the cochlea, and the hearing loss is more prominent in higher frequency range. This susceptibility of the basal turn of the cochlea is likely caused by a higher concentration of aminoglycoside at that structure than at the middle or apical turn, in a concentration-dependent manner (Dai et al., 2006; Dai and Steyger, 2008). Moreover, the susceptibility of the basal turn of the cochlea to ototoxic drugs can be explained by there being greater metabolic activity than at the apical turn of the cochlea (Sha et al., 2001). As shown in Figure 4, the OHCs damage caused by KM and furosemide and rescue effect of GV1001 were more prominent at the basal turn than the apical turn of the cochlea.

In this study, to assess the therapeutic effect of GV1001 against KM/furosemide-induced hair cell damage, we compared GV1001 (10 mg/kg) with dexamethasone (15 mg/kg) and saline by measuring the hearing thresholds and OHCs morphology. When compared with saline, the rescue effect of GV1001 was proven in all D0, D1 and D3 groups. As shown in Figures 2B, 4, the rescue effect of GV1001 seems to be superior to that of dexamethasone, but could not reach statistical significance in comparison of hearing thresholds and OHCs morphology between GV1001 and dexamethasone groups.

A previous study investigated the effect of different dexamethasone treatment times on OHCs survival following ototoxic insult with KM and furosemide. Mice pre-treated with dexamethasone (prior to 1 h of the insult) showed a statistically significant improvement in intact OHCs counts compared with the controls, but the mice subjected to dexamethasone post-treatment (at 1, 6, 12 and 72 h after the insult) showed highly variable OHCs counts (Fernandes and Lin, 2014). By contrast, in this study, GV1001 showed a rescue effect against OHCs damage even with delayed administration, i.e., at 3 days after the ototoxic insult.

The preventive effect of GV1001 pre-treatment against cochlear hearing loss and hair cell damage in a KM-induced deaf mouse model was verified in our previous study. As shown in Figure 1, all tested mice of the Day-1, Day-2 and Day-3 groups showed nearly complete hearing loss, but two out of three mice of the Day-1 group showed intact OHCs morphology. The cause of this discrepancy derived from phalloidin-stained at stereocilia of the OHCs. Two mice of the Day-1 group may have structurally intact OHCs morphology with loss of hearing function. The current study corroborated the rescue capacity of GV1001 against hair cell damage, as well as the ability to restore hearing even when administration was delayed by up to 3 days. Further study to investigate the underlying mechanisms of rescue effect of GV1001 will be needed.

In conclusion, we demonstrated that the cell-penetrating peptide GV1001 rescues hair cell damage and restores hearing in a KM/furosemide-induced deaf mouse model. Given that the rescue effect of GV1001 could be related to its anti-inflammatory, anti-oxidant, and anti-apoptotic activities, we predict that GV1001 could be useful not only in aminoglycoside-induced hearing loss, but also in acute cochlear hearing loss due to other causes. Because SNHL is caused by an imbalance in redox homeostasis and subsequent increase in ROS followed by the apoptotic pathway, GV1001 may have a potential clinical role in restoring hearing in cases of acute SNHL.

## Author Contributions

SHK analyzed data and wrote the manuscript. GJ performed the animal experiments and wrote the manuscript. SK provided technical assistance and wrote the manuscript. JWK designed the study and revised the manuscript. All authors read and approved the final manuscript.

## Conflict of Interest Statement

The authors declare that the research was conducted in the absence of any commercial or financial relationships that could be construed as a potential conflict of interest.
