# Supplementary material for: Novel Peptide Vaccine GV1001 Rescues Hearing in Kanamycin/Furosemide-Treated Mice
Source: Front Cell Neurosci. 2018 Jan 19;12:3. doi: 10.3389/fncel.2018.00003 (PMC5780435; doi:10.3389/fncel.2018.00003)
Supplement: Supplementary file 1 [file Table_1.doc]

**Supporting Information**

**Supplementary file S1** The numbers of experimental mice for experiment 1: assessment the initial temporal change of hearing and the extent of hair cell damage.

| Group  (Timing for sacrifice) | Total no. of mice | No. of dead mice | Final no. of mice |
| --- | --- | --- | --- |
| Day-1 | 3 | 0 | 3 |
| Day-2 | 3 | 0 | 3 |
| Day-3 | 3 | 1 | 2 |

**Supplementary file S2** The raw data of auditory brainstem response (ABR) threshold and outer hair cell (OHC) count in total 9 mice for experiment 1: assessment the initial temporal change of hearing and the extent of hair cell damage.

| Group | Animal no. | ABR threshold  at baseline | | | ABR threshold  at the date of sacrifice | | | OHC count  (no. of live cells/  no. of total cells) | | |
| --- | --- | --- | --- | --- | --- | --- | --- | --- | --- | --- |
|  |  | 8kHz | 16kHz | 32kHz | 8kHz | 16kHz | 32kHz | Apex | Middle | Base |
| Day-1 | 1 | 40 | 20 | 40 | 80 | 90 | 100 | 42/42 | 45/45 | 40/40 |
|  | 2 | 30 | 20 | 40 | 100 | 100 | 100 | 0/31 | 0/41 | 0/26 |
|  | 3 | 40 | 50 | 70 | 90 | 90 | 80 | 37/37 | 47/47 | 38/38 |
| Day-2 | 4 | 30 | 20 | 40 | 90 | 80 | 100 | 0/43 | 0/39 | 0/38 |
|  | 5 | 10 | 30 | 40 | 100 | 100 | 100 | 0/40 | 0/42 | 0/30 |
|  | 6 | 40 | 40 | 70 | 100 | 100 | 100 | 0/39 | 0/44 | 0/41 |
| Day-3 | 7 (death) |  |  |  |  |  |  |  |  |  |
|  | 8 | 20 | 20 | 70 | 100 | 100 | 100 | 0/38 | 0/43 | 0/46 |
|  | 9 | 30 | 30 | 70 | 100 | 100 | 100 | 0/39 | 0/42 | 0/43 |

**Supplementary file S3** The numbers of experimental mice for experiment 2: assessment the rescue effect of GV1001.

| Experiment (Administration period) | Group  (Drug) | Total no. of mice | No. of dead mice | Final no. of mice |
| --- | --- | --- | --- | --- |
| D0 | Saline | 10 | 1 | 9 |
|  | GV1001 | 10 | 1 | 9 |
|  | Dexamethasone | 10 | 0 | 10 |
| D1 | Saline | 10 | 1 | 9 |
|  | GV1001 | 10 | 2 | 8 |
|  | Dexamethasone | 10 | 2 | 8 |
| D3 | Saline | 10 | 1 | 9 |
|  | GV1001 | 10 | 1 | 9 |
|  | Dexamethasone | 10 | 1 | 9 |
| D7 | Saline | 10 | 2 | 8 |
|  | GV1001 | 10 | 2 | 8 |
|  | Dexamethasone | 10 | 2 | 8 |

**Supplementary file S4.** The raw data of auditory brainstem response (ABR) threshold, outer hair cell (OHC) count, and blood urea nitrogen (BUN) in total 120 mice for experiment 2: assessment the rescue effect of GV1001.

| Subgroup | Group | Animal no. | ABR threshold at baseline | | | ABR threshold at 1wk | | | ABR threshold at 2wk | | | OHC count  (no. of live cells/ no. of total cells) | | | BUN |
| --- | --- | --- | --- | --- | --- | --- | --- | --- | --- | --- | --- | --- | --- | --- | --- |
|  |  |  | 8kHz | 16kHz | 32kHz | 8kHz | 16kHz | 32kHz | 8kHz | 16kHz | 32kHz | Apex | Middle | Base |  |
| D0 | Saline | 1 | 30 | 50 | 70 | 100 | 100 | 100 | 100 | 100 | 100 | 0/33 | 0/45 | 0/39 | 28 |
|  |  | 2 | 20 | 20 | 60 | 90 | 90 | 100 | 100 | 100 | 100 | 0/39 | 0/43 | 0/35 | 17 |
|  |  | 3 | 20 | 30 | 40 | 90 | 90 | 100 | 100 | 90 | 100 | 0/40 | 0/38 | 0/35 | 27 |
|  |  | 4 | 30 | 20 | 30 | 100 | 90 | 100 | 100 | 90 | 100 | 0/33 | 0/40 | 0/15 | 14 |
|  |  | 5 | 40 | 30 | 60 | 100 | 100 | 100 | 100 | 100 | 100 | 0/37 | 0/45 | 0/30 | 27 |
|  |  | 6 (death) |  |  |  |  |  |  |  |  |  |  |  |  |  |
|  |  | 7 | 30 | 30 | 60 | 100 | 80 | 80 | 100 | 90 | 100 | 0/39 | 0/48 | 0/40 | 12 |
|  |  | 8 | 30 | 30 | 70 | 100 | 90 | 100 | 100 | 90 | 100 | 0/38 | 0/47 | 0/45 | 17 |
|  |  | 9 | 30 | 20 | 70 | 100 | 100 | 100 | 100 | 100 | 100 | 0/42 | 0/39 | 0/37 | 20 |
|  |  | 10 | 40 | 30 | 70 | 60 | 100 | 100 | 100 | 100 | 100 | 0/36 | 0/48 | 0/30 | 14 |
|  | GV1001 | 11 (death) |  |  |  |  |  |  |  |  |  |  |  |  |  |
|  |  | 12 | 30 | 20 | 70 | 40 | 70 | 40 | 40 | 30 | 80 | 32/32 | 41/41 | 29/33 | 14 |
|  |  | 13 | 40 | 40 | 40 | 20 | 30 | 30 | 40 | 30 | 40 | 32/32 | 41/41 | 30/30 | 28 |
|  |  | 14 | 40 | 30 | 50 | 30 | 20 | 50 | 40 | 20 | 50 | 39/39 | 38/38 | 43/43 | 15 |
|  |  | 15 | 20 | 20 | 80 | 40 | 30 | 50 | 40 | 20 | 70 | 38/38 | 36/36 | 40/40 | 17 |
|  |  | 16 | 40 | 30 | 40 | 100 | 100 | 100 | 100 | 100 | 100 | 0/36 | 36/41 | 35/37 | 27 |
|  |  | 17 | 30 | 30 | 50 | 80 | 80 | 100 | 100 | 80 | 100 | 0/45 | 0/40 | 0/31 | 13 |
|  |  | 18 | 30 | 20 | 60 | 100 | 60 | 90 | 30 | 20 | 70 | 42/42 | 40/40 | 38/38 | 17 |
|  |  | 19 | 30 | 20 | 30 | 40 | 20 | 40 | 100 | 100 | 100 | 0/45 | 0/40 | 0/32 | 18 |
|  |  | 20 | 30 | 20 | 40 | 30 | 20 | 50 | 40 | 30 | 50 | 31/31 | 51/51 | 36/36 | 18 |
|  | Dexamethasone | 21 | 20 | 30 | 70 | 40 | 30 | 60 | 30 | 20 | 70 | 40/40 | 42/42 | 40/40 | 20 |
|  |  | 22 | 40 | 20 | 80 | 40 | 20 | 60 | 40 | 70 | 80 | 41/41 | 40/40 | 44/44 | 17 |
|  |  | 23 | 30 | 20 | 70 | 100 | 100 | 100 | 30 | 60 | 70 | 44/44 | 38/38 | 45/45 | 20 |
|  |  | 24 | 40 | 20 | 50 | 40 | 20 | 80 | 30 | 30 | 60 | 39/39 | 43/43 | 45/45 | 21 |
|  |  | 25 | 30 | 20 | 30 | 70 | 50 | 80 | 50 | 30 | 80 | 43/43 | 36/36 | 39/39 | 17 |
|  |  | 26 | 30 | 20 | 70 | 100 | 100 | 100 | 100 | 80 | 90 | 0/42 | 0/30 | 0/35 | 19 |
|  |  | 27 | 30 | 20 | 60 | 100 | 100 | 100 | 100 | 100 | 100 | 0/45 | 0/46 | 0/34 | 13 |
|  |  | 28 | 30 | 20 | 60 | 100 | 90 | 100 | 100 | 100 | 100 | 0/44 | 0/40 | 0/45 | 13 |
|  |  | 29 | 40 | 20 | 60 | 100 | 100 | 100 | 100 | 100 | 100 | 0/38 | 0/43 | 36/36 | 14 |
|  |  | 30 | 30 | 20 | 40 | 40 | 30 | 60 | 30 | 20 | 70 | 44/44 | 43/43 | 42/42 | 18 |
| D1 | Saline | 31 | 30 | 20 | 20 | 40 | 40 | 30 | 60 | 70 | 90 | 35/35 | 0/33 | 0/31 | Not tested |
|  |  | 32 | 20 | 20 | 30 | 100 | 70 | 80 | 100 | 100 | 100 | 0/43 | 0/38 | 0/45 | Not tested |
|  |  | 33 | 20 | 20 | 40 | 100 | 100 | 100 | 90 | 100 | 100 | 0/30 | 0/20 | 0/30 | Not tested |
|  |  | 34 | 20 | 20 | 20 | 90 | 100 | 100 | 90 | 100 | 100 | 0/36 | 0/38 | 0/40 | Not tested |
|  |  | 35 | 30 | 30 | 70 | 70 | 90 | 80 | 100 | 100 | 100 | 0/40 | 0/40 | 0/15 | Not tested |
|  |  | 36 | 30 | 20 | 20 | 80 | 100 | 100 | 90 | 90 | 100 | 0/45 | 0/45 | 0/46 | Not tested |
|  |  | 37 | 40 | 20 | 30 | 90 | 90 | 90 | 90 | 90 | 90 | 0/40 | 0/43 | 0/39 | Not tested |
|  |  | 38 | 30 | 30 | 40 | 100 | 100 | 100 | 100 | 100 | 100 | 0/40 | 0/43 | 0/15 | Not tested |
|  |  | 39 | 30 | 30 | 40 | 100 | 100 | 100 | 100 | 100 | 100 | 0/42 | 0/40 | 0/37 | Not tested |
|  |  | 40 (death) |  |  |  |  |  |  |  |  |  |  |  |  |  |
|  | GV1001 | 41 | 20 | 30 | 30 | 100 | 100 | 90 | 100 | 100 | 70 | 0/39 | 0/45 | 0/45 | Not tested |
|  |  | 42 | 40 | 40 | 40 | 40 | 30 | 60 | 30 | 20 | 40 | 45/45 | 45/45 | 36/36 | Not tested |
|  |  | 43 | 30 | 30 | 60 | 50 | 30 | 50 | 30 | 20 | 60 | 45/45 | 35/35 | 36/36 | Not tested |
|  |  | 44 | 30 | 20 | 30 | 90 | 100 | 100 | 100 | 100 | 100 | 0/30 | 0/37 | 0/48 | Not tested |
|  |  | 45 | 30 | 30 | 20 | 30 | 30 | 40 | 40 | 30 | 50 | 45/45 | 45/45 | 40/40 | Not tested |
|  |  | 46 | 30 | 30 | 30 | 20 | 30 | 40 | 30 | 30 | 40 | 39/39 | 40/40 | 25/25 | Not tested |
|  |  | 47 (death) |  |  |  |  |  |  |  |  |  |  |  |  |  |
|  |  | 48 | 30 | 30 | 50 | 30 | 40 | 70 | 30 | 30 | 60 | 39/40 | 45/45 | 40/40 | Not tested |
|  |  | 49 | 30 | 20 | 30 | 100 | 90 | 100 | 90 | 100 | 100 | 0/45 | 0/45 | 0/20 | Not tested |
|  |  | 50 (death) |  |  |  |  |  |  |  |  |  |  |  |  |  |
|  | Dexamethasone | 51 | 50 | 40 | 30 | 40 | 30 | 30 | 30 | 20 | 20 | 43/43 | 44/45 | 38/38 | Not tested |
|  |  | 52 | 30 | 40 | 40 | 100 | 100 | 100 | 100 | 100 | 100 | 0/40 | 0/20 | 0/35 | Not tested |
|  |  | 53 | 30 | 30 | 30 | 90 | 100 | 100 | 90 | 100 | 100 | 40 | 0/40 | 0/40 | Not tested |
|  |  | 54 | 30 | 30 | 20 | 90 | 90 | 100 | 90 | 90 | 90 | 0/45 | 0/45 | 0/42 | Not tested |
|  |  | 55 | 40 | 30 | 30 | 70 | 90 | 90 | 100 | 100 | 100 | 0/23 | 0/40 | 0/20 | Not tested |
|  |  | 56 (death) |  |  |  |  |  |  |  |  |  |  |  |  |  |
|  |  | 57 | 40 | 30 | 30 | 40 | 30 | 30 | 20 | 30 | 60 | 40/40 | 46/46 | 39/39 | Not tested |
|  |  | 58 | 30 | 20 | 30 | 30 | 30 | 30 | 30 | 30 | 30 | 39/39 | 40/40 | 38/38 | Not tested |
|  |  | 59 | 30 | 20 | 30 | 100 | 90 | 100 | 100 | 80 | 100 | 0/39 | 0/41 | 0/39 | Not tested |
|  |  | 60 (death) |  |  |  |  |  |  |  |  |  |  |  |  |  |
| D3 | Saline | 61 | 20 | 20 | 40 | 100 | 100 | 100 | 90 | 100 | 100 | 0/45 | 0/42 | 0/41 | 20 |
|  |  | 62 | 20 | 20 | 20 | 100 | 90 | 90 | 100 | 100 | 100 | 0/44 | 0/43 | 0/50 | 14 |
|  |  | 63 | 30 | 20 | 30 | 100 | 100 | 90 | 100 | 90 | 90 | 19/33 | 5/42 | 0/30 | 17 |
|  |  | 64 | 20 | 20 | 80 | 100 | 80 | 100 | 100 | 100 | 100 | 0/30 | 0/40 | 0/40 | 18 |
|  |  | 65 | 20 | 20 | 50 | 100 | 100 | 100 | 100 | 100 | 100 | 0/43 | 0/42 | 0/43 | 20 |
|  |  | 66 | 30 | 20 | 70 | 100 | 100 | 100 | 100 | 100 | 100 | 5/45 | 0/42 | 0/32 | 25 |
|  |  | 67 (death) |  |  |  |  |  |  |  |  |  |  |  |  |  |
|  |  | 68 | 40 | 20 | 50 | 100 | 100 | 100 | 100 | 100 | 100 | 0/39 | 0/45 | 0/42 | 20 |
|  |  | 69 | 30 | 20 | 60 | 90 | 100 | 100 | 100 | 100 | 100 | 0/40 | 0/42 | 0/30 | 16 |
|  |  | 70 | 40 | 20 | 60 | 100 | 90 | 80 | 100 | 100 | 100 | 0/35 | 0/45 | 0/40 | 17 |
|  | GV1001 | 71 | 20 | 20 | 60 | 40 | 40 | 80 | 40 | 20 | 60 | 51/51 | 40/40 | 45/45 | 12 |
|  |  | 72 | 30 | 30 | 30 | 20 | 20 | 40 | 30 | 20 | 20 | 42/42 | 45/46 | 36/36 | 18 |
|  |  | 73 | 30 | 30 | 40 | 40 | 50 | 70 | 30 | 30 | 60 | 39/39 | 48/48 | 38/38 | 13 |
|  |  | 74 | 30 | 30 | 40 | 90 | 90 | 100 | 80 | 70 | 100 | 0/36 | 0/36 | 0/35 | 18 |
|  |  | 75 (death) |  |  |  |  |  |  |  |  |  |  |  |  |  |
|  |  | 76 | 30 | 30 | 50 | 20 | 30 | 50 | 30 | 30 | 60 | 41/41 | 43/43 | 18/18 | 21 |
|  |  | 77 | 40 | 20 | 40 | 100 | 100 | 100 | 100 | 100 | 100 | 21/39 | 0/38 | 0/35 | 21 |
|  |  | 78 | 40 | 20 | 50 | 100 | 90 | 100 | 100 | 90 | 100 | 0/42 | 0/38 | 0/42 | 16 |
|  |  | 79 | 40 | 50 | 70 | 100 | 100 | 100 | 100 | 100 | 100 | 0/42 | 0/33 | 0/30 | 17 |
|  |  | 80 | 30 | 30 | 50 | 30 | 40 | 80 | 30 | 40 | 80 | 39/39 | 41/41 | 34/34 | 17 |
|  | Dexamethasone | 81 | 20 | 20 | 30 | 90 | 80 | 70 | 90 | 80 | 90 | 0/36 | 0/32 | 0/30 | 19 |
|  |  | 82 | 30 | 30 | 60 | 100 | 100 | 100 | 100 | 90 | 80 | 0/43 | 0/34 | 0/40 | 33 |
|  |  | 83 | 30 | 20 | 30 | 100 | 100 | 100 | 100 | 100 | 90 | 0/34 | 0/41 | 0/43 | 29 |
|  |  | 84 | 30 | 30 | 50 | 60 | 30 | 100 | 80 | 60 | 100 | 42/42 | 40/40 | 47/47 | 14 |
|  |  | 85 (death) |  |  |  |  |  |  |  |  |  |  |  |  |  |
|  |  | 86 | 30 | 30 | 70 | 100 | 80 | 90 | 100 | 90 | 100 | 0/36 | 0/44 | 0/45 | 30 |
|  |  | 87 | 20 | 20 | 50 | 100 | 90 | 100 | 100 | 90 | 100 | 3/42 | 0/43 | 0/40 | 22 |
|  |  | 88 | 50 | 20 | 50 | 100 | 100 | 100 | 100 | 100 | 100 | 0/43 | 0/42 | 0/43 | 25 |
|  |  | 89 | 30 | 40 | 90 | 90 | 80 | 90 | 100 | 100 | 100 | 0/40 | 0/41 | 0/38 | 26 |
|  |  | 90 | 30 | 30 | 50 | 100 | 100 | 100 | 50 | 40 | 80 | 38/38 | 45/49 | 40/40 | 23 |
| D7 | Saline | 91 | 40 | 40 | 60 | 100 | 100 | 100 | 100 | 100 | 100 | 0/39 | 0/36 | 0/42 | 22 |
|  |  | 92 | 50 | 40 | 80 | 100 | 100 | 100 | 100 | 100 | 100 | 0/36 | 0/32 | 0/13 | 23 |
|  |  | 93 (death) |  |  |  |  |  |  |  |  |  |  |  |  |  |
|  |  | 94 | 50 | 20 | 50 | 90 | 100 | 100 | 100 | 90 | 100 | 0/39 | 0/41 | 0/40 | 17 |
|  |  | 95 | 50 | 30 | 80 | 100 | 100 | 100 | 100 | 100 | 100 | 0/35 | 0/44 | 0/46 | 13 |
|  |  | 96 | 20 | 40 | 80 | 100 | 100 | 100 | 80 | 100 | 90 | 0/39 | 0/45 | 0/40 | 13 |
|  |  | 97 | 40 | 30 | 60 | 100 | 100 | 100 | 100 | 100 | 100 | 0/38 | 0/44 | 0/33 | 15 |
|  |  | 98 | 30 | 30 | 40 | 100 | 100 | 100 | 100 | 100 | 100 | 0/42 | 0/38 | 0/39 | 19 |
|  |  | 99 | 30 | 20 | 60 | 100 | 100 | 100 | 100 | 100 | 100 | 0/40 | 0/42 | 0/32 | 18 |
|  |  | 100 (death) |  |  |  |  |  |  |  |  |  |  |  |  |  |
|  | GV1001 | 101 | 50 | 40 | 80 | 100 | 80 | 100 | 100 | 100 | 100 | 0/40 | 0/42 | 0/18 | 20 |
|  |  | 102 | 40 | 30 | 70 | 100 | 100 | 100 | 100 | 100 | 100 | 0/33 | 0/40 | 0/17 | 19 |
|  |  | 103 | 40 | 30 | 60 | 100 | 100 | 100 | 100 | 100 | 100 | 0/38 | 0/42 | 0/34 | 12 |
|  |  | 104 | 40 | 30 | 40 | 100 | 100 | 100 | 100 | 100 | 100 | 0/41 | 0/43 | 0/39 | 17 |
|  |  | 105 (death) |  |  |  |  |  |  |  |  |  |  |  |  |  |
|  |  | 106 | 40 | 40 | 80 | 100 | 100 | 100 | 100 | 100 | 100 | 0/43 | 0/48 | 0/42 | 18 |
|  |  | 107 | 40 | 40 | 70 | 100 | 100 | 100 | 100 | 100 | 100 | 0/39 | 0/45 | 0/29 | 23 |
|  |  | 108 | 40 | 30 | 50 | 100 | 100 | 100 | 100 | 100 | 100 | 0/41 | 0/49 | 0/25 | 22 |
|  |  | 109 (death) |  |  |  |  |  |  |  |  |  |  |  |  |  |
|  |  | 110 | 30 | 30 | 60 | 100 | 100 | 100 | 100 | 100 | 100 | 0/42 | 0/45 | 0/15 | 20 |
|  | Dexamethasone | 111 | 30 | 20 | 60 | 100 | 100 | 100 | 100 | 100 | 100 | 0/36 | 0/42 | 0/28 | 28 |
|  |  | 112 | 40 | 20 | 60 | 100 | 100 | 100 | 100 | 100 | 100 | 0/45 | 0/24 | 0/39 | 14 |
|  |  | 113 | 40 | 20 | 60 | 100 | 100 | 100 | 100 | 100 | 100 | 0/42 | 0/41 | 0/15 | 18 |
|  |  | 114 (death) |  |  |  |  |  |  |  |  |  |  |  |  |  |
|  |  | 115 | 40 | 20 | 30 | 100 | 100 | 100 | 100 | 100 | 100 | 0/40 | 0/44 | 0/30 | 17 |
|  |  | 116 | 30 | 30 | 60 | 100 | 100 | 100 | 100 | 100 | 100 | 0/38 | 0/46 | 0/24 | 26 |
|  |  | 117 | 30 | 30 | 60 | 100 | 100 | 100 | 100 | 80 | 90 | 0/39 | 0/46 | 0/43 | 21 |
|  |  | 118 | 40 | 30 | 70 | 100 | 100 | 100 | 100 | 100 | 100 | 0/42 | 0/40 | 0/47 | 17 |
|  |  | 119 | 40 | 40 | 40 | 100 | 100 | 100 | 100 | 100 | 100 | 0/33 | 0/42 | 0/44 | 18 |
|  |  | 120 (death) |  |  |  |  |  |  |  |  |  |  |  |  |  |
